# Effectiveness of Millet–Pulse–Groundnut Based Formulations in Improving the Growth of Pre-School Tribal Children in Telangana State, India

**DOI:** 10.3390/nu16060819

**Published:** 2024-03-13

**Authors:** Datta Mazumdar Saikat, Afari-Sefa Victor, Selvaraj Aravazhi, Durgalla Priyanka, Seetha Anitha, Nedumaran Tamilselvi, Gaddam Divya Nancy, Mane Harshvardhan, Bhattacharjee Suchiradipta, Swamikannu Nedumaran, Raman Anitha, Banerjee Roopa, Padmanabhan Jyosthnaa, Bose Disha

**Affiliations:** International Crops Research Institute for the Semi-Arid Tropics (ICRISAT), Hyderabad 502324, India; victor.afarisefa@icrisat.org (A.-S.V.); aravazhi.selvaraj@worldveg.org (S.A.); priyanka.durgalla@icrisat.org (D.P.); dr.anithaseetha@gmail.com (S.A.); tamilselvi.nedumaran@icrisat.org (N.T.); divya.nancy@icrisat.org (G.D.N.); harshvardhan.mane@icrisat.org (M.H.); b.suchiradipta@gmail.com (B.S.); swamikannu.nedumaran@icrisat.org (S.N.); anitha.raman@icrisat.org (R.A.); roopa.banerjee@icrisat.org (B.R.); jyosthnaa.padmanabhan@icrisat.org (P.J.); disha.bose@icrisat.org (B.D.)

**Keywords:** nutrition intervention, millets, sorghum, ready-to-eat food, ready-to-cook food, pre-school children

## Abstract

A community-level nutritional intervention was implemented among tribal children (3 to 6 years of age) in Telangana, India. The one-year intervention involved six nutrient-rich formulations of millet–pulse–groundnut-based products suited to local taste preferences. Anthropometric measurements of height, weight, and mid-upper-arm circumference (MUAC) along with haemoglobin (Hb) levels were monitored at baseline and endline. The treatment group showed considerable gains in height (3.2 cm), weight (1.68 kg), and MUAC (0.33 cm) over the control group. The paired *t*-test indicated significant differences (*p* < 0.01) between the pre- and post-intervention anthropometric measurements. Positive shifts were observed in terms of wasting (WHZ; −1.2 ± 1.3 to −0.9 ± 1), stunting (HAZ; −1.8 ± 1.6 to −0.3 ± 1.3), and underweight (WAZ; −1.9 ± 1.2 to −0.7 ± 1) in the treatment group. The Hb levels in the treatment group also improved significantly from 9.70 ± 0.14 g/dL (moderately anaemic) to 11.08 ± 0.13 g/dL (non-anaemic). Post-intervention focus group discussions (FGDs) involving mothers and teachers confirmed these positive impacts. Thus, a nutritional intervention formulated using climate-resilient millets, pulses, and groundnuts promotes dietary diversity and improves the nutrition and health statuses of children.

## 1. Introduction

Undernutrition (wasting, stunting, and underweight), micronutrient deficiency, and other health-related issues are immense public health challenges that plague developing countries in particular. Though other methods for addressing undernutrition exist, such as fortification and supplementation, traditional natural food-based dietary interventions seem to be the most sustainable option to confront this challenge. The EAT-Lancet Commission report builds upon the concept of planetary health and has put forth the new term “Planetary health diet”. It highlights the critical role that diets play in linking human health and environmental sustainability, as well as the need to integrate both into a common global agenda for food system transformation to achieve its Sustainable Development Goals (SDGs) [1]. The “Planetary health diet” suggests increasing the intake of whole grains, vegetables, fruits, legumes, and pulses, which are currently consumed considerably below global target levels [2]. According to the World Health Organization (WHO), a healthy diet is recommended to include 400 g (five portions) of fruits and vegetables every day [3]. Among thousands of indigenous food crops existing on this planet, rice, wheat, and maize are the three staple foods majorly consumed by humans and provide 60% of our calories [4]. Thus, diversifying staple foods is an important step towards achieving maximum nutritional benefits, as staples form the largest portion of our diets. This poses the following question: Which other alternate staples could fit into our diets to provide the required energy and maximum nutritional availability? 

Millets, pulses, and groundnuts are traditional crops grown in Asia and Africa and are rich sources of macro- and micronutrients [5,6]. However, their share in the consumer food basket is limited. Scientific evidence proves that millets have the potential to reduce type 2 diabetes, manage lipid profiles, and reduce anaemia [7]. Millets contain sulphur-rich amino acids, such as methionine and cysteine, and are generally low in lysine; hence, they can contribute to a balanced diet when consumed with protein-rich pulses such as pigeon peas and chickpeas, which are deficient in sulphur-containing amino acids but rich in lysine [6]. Finger millet is a rich source of calcium, an important mineral for child growth and bone health [8]. Moreover, groundnuts are also one of the most energy-dense food sources, providing 520 kcal energy per 100 gm [6].

Millets, also called nutri-cereals, have the potential to play a crucial role in combatting food insecurity and malnutrition [9]. A community feeding pilot study conducted among school-going children in India proved that millet-based food formulations are not only acceptable, but also help to improve the growth of children compared to regularly consumed rice-based diets. During this study, millets were introduced in the form of common South Indian food items, namely, idli, khichdi, upma, and bisibella bath (traditional local rice-based recipes), in which rice was replaced by millet. Dhanashakti, a biofortified pearl millet that is high in iron and zinc, was used for the recipe formulations. In addition to millets, legumes and vegetables were added to further elevate the nutrient profile of the menu, which helped to improve the health statuses of the consumers. The Government of India supports the supplementary nutritional needs of children (below 6 years) as part of the Integrated Child Development Services (ICDS) scheme under the component Supplementary Nutrition Programme (ICDS-SNP) [10]. In addition, it also supports the supplementary nutritional needs of pregnant and lactating mothers. The ICDS-SNP scheme is primarily designed to bridge the gap between Recommended Dietary Allowance (RDA) and Average Daily Intake (ADI).

Pre-school children (3 to 6 years) can visit Anganwadi Centres (AWCs—community feeding centres under ICDS) and receive food under the ICDS-SNP. The food is intended to provide 500 calories of energy and 12–15 g of protein per child per day. Since a child of this age group is not capable of consuming a meal of 500 calories in one sitting, the guidelines prescribe the provision of a morning snack in the form of milk/banana/seasonal fruits/micronutrient-fortified food, etc., and a hot, cooked mid-day meal. The ICDS-SNP school meal is critical for children, especially those in the tribal regions of India, as it provides a major portion of their food and nutritional needs.

However, the major staple served at school, as part of the mid-day meal, is refined rice. The Government of India guidelines recommend providing a hot meal of rice (75 g), dal (soup prepared using 15 g of red gram), vegetables (25 g), egg (50 g), and nutri-snacks/channa dal (split and roasted chickpea). Thus, the mid-day meal recipe is designed to provide a total energy of 548 kcal per day for children between 3 and 6 years of age at AWCs [11].

Rice is majorly consumed by people in India, as it is supplied at a subsidised rate through the Public Distribution System (PDS). Based on the evidence on the health benefits of consuming millets and considering the need to tackle increasing malnutrition and climate change issues, the Government of India has recommended introducing millets into school meals [11].

The poor health outcomes among the tribal population in India, particularly among women and children, are attributed to the lack of dietary diversity. Financial circumstances, shifts to lucrative crops, dependency on a public distribution system (PDS) that failed to incorporate traditional food patterns, etc., have played a role in the loss of dietary diversity among tribal people [12]. Reports based on the fourth round of the National Family Health Survey (NFHS 4, 2015–2016) highlighted the alarmingly poor nutritional status among women and children in the tribal areas of Telangana State, India. The government of Telangana State, in collaboration with ICRISAT and other state government departments such as the Integrated Tribal Development Agency (ITDA), designed an initiative to address these prevalent nutrition issues among tribal people in the state. The initiative is intended to improve the health outcomes among children under 6 years as well as anaemic women in the age group of 15–49 years. The Agribusiness and Innovation Platform (AIP) of ICRISAT collaborated with the Department of Tribal Welfare (TWD) and the Department of Women Development and Child Welfare (WDCW) of Telangana in conceptualising, formulating, and producing nutritious millet–pulse–groundnut-based food products with the objective of providing affordable, safe, and nourishing foods to the tribal people.

Thus, Giri Poshana [Giri (Tribal) Poshana (nutrition)], was conceived as a tribal nutrition project with the aim of providing nutritional support to children (3–6 years) and tribal women (pregnant women and lactating mothers) at the AWCs, involving ITDAs. Giri Poshana focussed on two of the aspirational districts [13], Bhadadri Kothagudam and Jayashankar Bhupalpally (including Mulugu), in India’s Telangana State [13].

Government reports clearly indicate the abysmal health status of the tribal population of India, specifically in Telangana State. However, efforts were not made in the interest of enhancing the nutritional levels of this population. As a first measure, the state government of Telangana proposed this intervention for tribal children. Moreover, although research exists on the myriad health benefits of millets and pulses in the field of diet and nutrition, empirical evidence to support these claims are rare, particularly in the Indian context. Thus, this study contributes to the literature in two ways:

1. It focusses on the health parameters of tribal children, which have often been overlooked in the existing literature;
2. It provides empirical evidence on the effects of introducing millet–pulse–groundnut-based products in diets on the salient health parameters of children.

Considering the challenges of rapidly changing climate conditions and prevailing malnutrition, especially among the tribal population, the alternate climate-resilient staples, namely, millets with legumes and/or pulses, have the potential to provide sustainable solutions to combat the environmental stress of climate change alongside ensuring food and nutritional security [14]. The current study was conducted with the aim of determining the impact of introducing the millet–pulse–groundnut-based Ready-to-Cook (RTC) meals and Ready-to-Eat (RTE) snacks, as an addition to the regular ICDS-SNP mid-day school meal, on the acceptability, growth (height, weight, and MUAC), and Hb levels among pre-school tribal children attending AWCs in the ITDA areas of Bhadrachalam and Eturunagaram in the Telangana State of India. 

The hypothesis that the research seeks to answer is as follows:

**Hypothesis** **1:** *Millet–pulse–groundnut-based food items can improve health parameters such as height, weight, mid-upper arm circumference, and haemoglobin levels among the target population from baseline to endline*.

Our design of the intervention helps us explore this hypothesis.

## 2. Materials and Methods

### 2.1. Ethical Approval

ICRISAT’s Institutional Review Board (IRB) is the body responsible for ensuring that all research involving “Humans” is carried out in an ethical manner. The said study received ethical approval from ICRISAT’s IRB. The details of the ethical clearance are as follows: (a) project identification code: IEC-ICRISAT/20210707/07; (b) date of approval: 7 July 2021; (c) name of the ethics committee: Institutional Review Board (IRB), International Crops Research Institute for the Semi-Arid Tropics (ICRISAT).

### 2.2. Methodology

Prior to the study, a sensory evaluation of the formulated millet–pulse–groundnut-based food products was conducted among selected target beneficiaries to assess the acceptability and preferences. A representative sample of children (3–6 years of age) was selected in each interventional ITDA area. The selected sample was provided with food products, and feedback was recorded through a specifically designed questionnaire using a five-point hedonic rating scale (1 = dislike very much, 2 = dislike slightly, 3 = neither like nor dislike, 4 = like slightly, 5 = like very much) (Table S1). Based on the stated responses and preferences, the products were re-formulated, the formulation was finalised, and the products were distributed among the beneficiaries at the selected AWCs. The distribution and feeding were undertaken with the help of the AWC staff prior to the COVID-19 pandemic. With the onset of the COVID-19 pandemic, the restrictions on movements and closure of the AWCs necessitated a change in the product formats (from RTC to RTE), as the food products needed to be distributed as take-home rations (THRs) directly to the beneficiary households. The challenges posed by the pandemic and other natural disasters, such as floods in the intervention areas, caused intermittent breakdowns in the distribution of the food products, resulting in an extended duration of the project period from 12 to 18 months. However, the feeding intervention was only for 12 months. At the end of the intervention period (endline), anthropometry and Hb data were collected by trained enumerators to assess changes in the nutritional status among the children by comparing with measurements recorded before the intervention (baseline).

The anthropometric and Hb data were further complemented with qualitative data collected through 13 FGDs and 26 key informant interviews with AWC teachers (Questionnaire S1), where the outcomes of the nutritional intervention were discussed. Standard protocols were followed during the conduct of the FGDs with the AWC teachers, pre-school children, and mothers in order to understand their (i) awareness of millets; (ii) traditional food habits and consumption; (iii) changes in consumption pattern; (iv) post-intervention shifts in consumption of millet–pulse–groundnut-based products; (v) any improvements observed in the school attendance among children; and (vi) portion sufficiency of the products distributed.

### 2.3. Administrative Structure of the Study Sites and Sample Sizes

**Figure 1:**
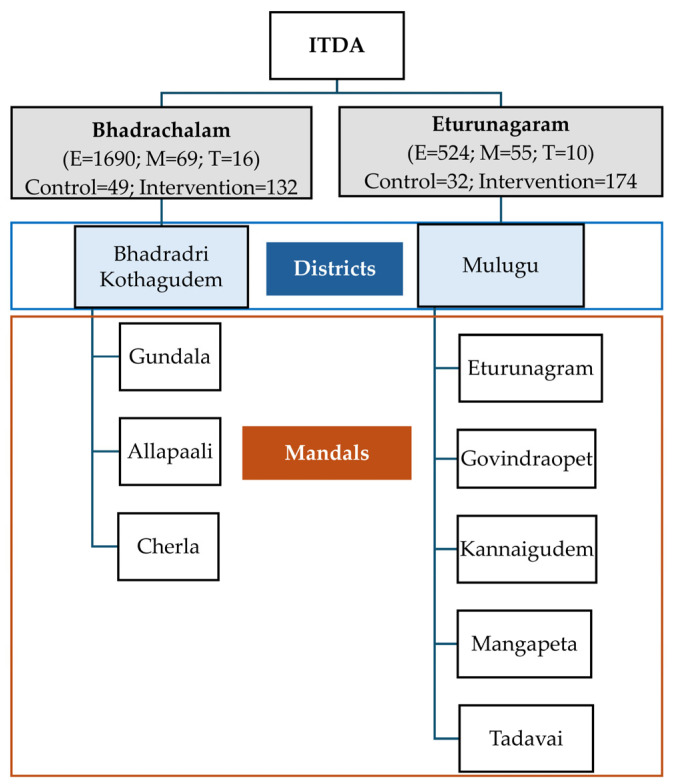
Flowchart of administrative divisions of study areas in Eturunagaram and Bhadrachalam ITDAs. Location: Anthropometry measurements, FGDs, and key informant interviews were conducted at Anganwadi centres in the respective areas. E—Preschool children enrolled in the Giri Poshana programme in target ITDAs; M—Mothers of the pre-school children who participated in the FGDs; T—Anganwadi teachers interviewed; Control—Children who did not receive millet feed and were followed up for their anthropometric measurements for the entire duration of the study; Intervention—Children who received millet feed and were followed up for their anthropometric measurements for the entire duration of the study; E, M, T, control, and intervention are the total number of individuals across three and five mandals (M) in the two districts (D).

Telangana State in India is divided into “Districts”, which are again classified into “Sub-divisions”. The sub-divisions are further divided into “Mandals”, and the mandals consist of villages [15]. Dense tribal areas, namely, Bhadrachalam and Eturunagaram ITDAs involving 273 AWCs, were selected by the government body for the dietary intervention comprising millet–pulse–groundnut-based products (Figure 1). The sample size of this study included 306 pre-schoolers who consumed the millet–pulse–groundnut-based diet (treatment group), and 81 pre-school children who did not receive the intervention formed the control group.

**Figure 2:**
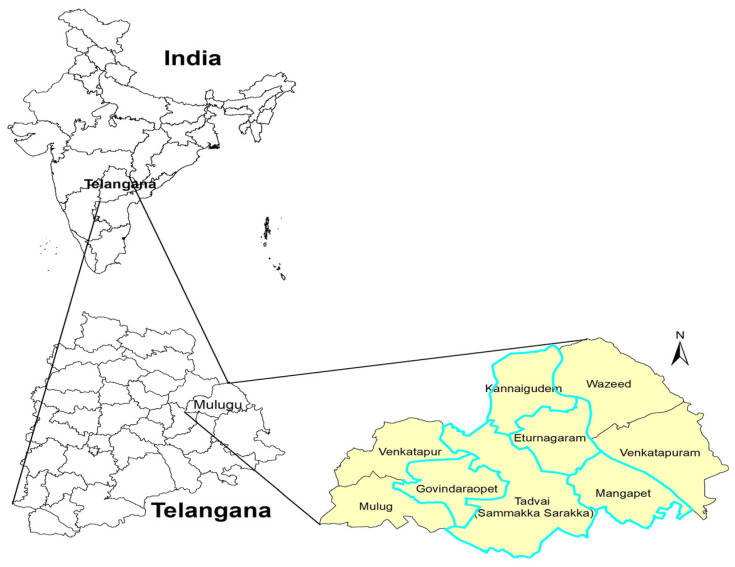
ITDA-Eturunagaram showing mandals involved in the study.

**Figure 3:**
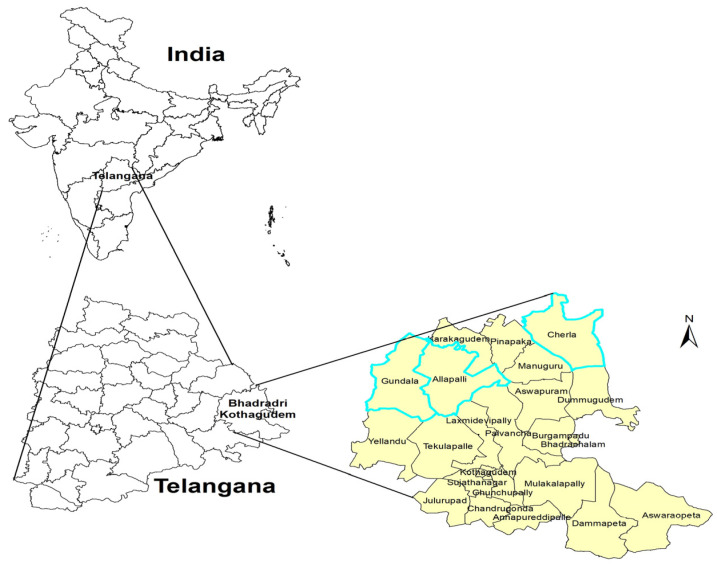
ITDA-Bhadrachalam showing mandals involved in the study.

For the Bhadrachalam ITDA area, three mandals were chosen, namely Gundala, Allapally, and Cherla. Within each of these mandals, at least two FGDs were carried out. At the Eturunagaram ITDA, five mandals were selected: Govidraopet, Kannaigudem, Mangapet, Thadvai, and Eturunagaram. One FGD was conducted in each of these mandals except for Mangapet, where two FGDs were conducted (Figure 2 and Figure 3).

Each FGD consisted of 10–12 mothers of pre-school children who were selected from the designated AWCs. The total number of mothers who participated in the FGDs conducted in Bhadrachalam and Eturunagaram ITDAs were 69 and 55, respectively. Additionally, AWC teachers from ITDA-Bhadrachalam (16) and ITDA-Eturunagaram (10) participated in individual interviews.

### 2.4. Products Used in Giri Poshana and Their Nutrient Profiles

The food products used for the nutritional intervention were formulated using local climate-resilient crops such as millets, pulses, groundnuts, etc., which provide energy, protein, fat, and micronutrients. The intervention included RTC products such as sorghum (jowar) meal (sorghum, Bengal gram, groundnut, spices and condiments), multigrain savoury meal (sorghum, foxtail millet, green gram with spices and condiments), and multigrain sweet meal (sorghum, wheat, almond, raisin, sugar, and jaggery). These were given in the form of cooked meals (per serving per day was 50 g of uncooked meal equivalent to 150 g post cooking). The RTE snacks comprised an energy bar (groundnut, jaggery, sesame, liquid glucose, and sugar), sorghum bytes (sorghum, maize, rice, soya, oil, seasoning mix), and nutri-cookies (sorghum, finger millet, soya, vegetable fat, sugar, custard powder, cinnamon, and raising agent) provided in quantities of 35 g per serving per day as a combination of a breakfast meal and evening snack for 6 days in a week, as per the standardised menu.

The nutrient profiling (Table 1) was undertaken using approved Association of Agricultural Chemists (AOAC) International methods for each of the respective parameters.

The products were designed to diversify and supplement the existing diets of the target population and were not fortified with additional micronutrients. The millets used in the food product formulations were malted to enhance the digestibility of carbohydrate/proteins and to ensure reductions in anti-nutrients, thus leading to increased micronutrient bioavailability [16]. The AWC teachers were sensitised to cooking methods for the RTC products, serving portions, and the nutritional benefits of the products, along with awareness of basic hygiene practices to be followed during food preparation, storage, and serving. 

### 2.5. Focus Group Discussions (FGDs)

The mothers of the pre-school (3–6 years) children and the teachers from the AWCs were included in the FGDs. The respondents were interviewed individually to assess the acceptance, preferences, and challenges faced with respect to the three RTC and three RTE products distributed to the beneficiaries during the intervention period.

These FGD sessions were conducted until the discussions or questions reached a saturation point, with the time varying between 60 and 90 min. A facilitator guided the group through the discussions in the local language, while a note-taker documented the conversation without participating in the interactions, and another team member recorded the conversation to create a transcript of the event. 

The responses on acceptability of the six products were recorded using a 6-point scale of 1 to 6, with 6 being highly accepted and 1 being least accepted. The FGDs were designed to elicit responses about awareness of millets, the challenges faced while using them at home, and their choices with respect to the continued use of the products at AWCs or in homes. Some of the specific questions asked to the teachers during individual interviews were related to challenges of implementation and changes observed among the children’s cognitive behaviour and overall performance.

### 2.6. Data Collection and Analysis

Anthropometry data that included indicators of height-for-age (stunting), weight-for-height (wasting), weight-for-age (underweight), and MUAC were collected from both the treatment group (n = 306) and the control group (n = 81) of pre-school children at both baseline and endline. The instruments used included a stadiometer (IS Indosurgicals Stadiometer—height), weighing scale (Seca 813 high-capacity digital flat scale—weight), and MUAC tape (IS Indosurgicals Muac Tape—MUAC). The anthropometric indicators were analysed using a WHO-developed online tool (WHO Anthro Survey Analyser, version 3.2.2). The paired *t*-test was used to assess the mean anthropometric differences between the treatment and control groups at baseline and endline, and it was performed using Stata 14^®^ software. Total Hb data were collected from children using a non-invasive haemoglobin measuring device, Masimo Rainbow SET^®^ Pulse Co-Oximetry, in both the intervention and control groups at baseline and endline.

#### 2.6.1. Data Preprocessing for Haemoglobin DATA Analysis

Hb observations were measured using a non-invasive device, “Masimo Rainbow SET^®^ Pulse Co-Oximetry” (Masimo Corporation, Irvine, CA, USA, sourced from India office Masimo Medical Technologies India Pvt. Ltd.), and they were first corrected in accordance with the reference “invasive” method using the following equation, which was derived based on Taffé and Taffé et al. (2020) [17]:

$${corrected\;Hb}_{non-invasive}=\frac{{measured\;Hb}_{non-invasive}-6.6082}{0.4865}$$

The reference method, which is the ‘gold standard,’ is not error-free but is almost free from measurement errors. What matters is the amount by which the two methods disagree [18], which is referred to as the bias. Total Hb levels were measured for 306 individuals in the treatment group while 81 individuals were monitored in the control group. However, two individuals from the treatment and one from the control group were excluded because the baseline and endline differences doubled after bias corrections with reference to the invasive method. Apart from this, 19 individuals in the treatment group did not have baseline observations.

#### 2.6.2. Repeated Measures Analysis of Variance (RM-ANOVA)

This is a classical repeated measures design in time, where experimental units (individuals) receive a treatment and multiple measurements are made on the same individual for the response variable at different time points (baseline and endline). Thus, the observations in the data set are no longer assumed to be independent, as the residual errors are correlated among time points. The mean change in the Hb levels when moving from baseline to endline in the control and treatment groups is directly measured by the Time × Treatment interaction term in the repeated measures ANOVA.

Statistical model:

The ANOVA model for repeated Hb measures in time is as follows:

$$Hb=mean+group+group\times time+ind\left(group\right)+error$$

where ind(group) indicates individuals within a group. 

However, the residual (an estimate of the error) is a matrix, as follows:

$$R=\left(\begin{array}{cc} \sigma_{1}^{2} & \sigma_{12}\\ \sigma_{12} & \sigma_{2}^{2} \end{array}\right)$$

In the residual matrix R, the diagonals $\sigma_{1}^{2}$ and $\sigma_{2}^{2}\;$ are the residual variances for the baseline and endline, respectively, and the off-diagonals are the covariances between the two time-points, assumed to be the same across all individuals. The Time main effect was not included in the model, as the overall effect of time across the two groups was not the focus.

## 3. Results

### 3.1. Pre-Intervention Assessment of Preferences

The study conducted prior to the commencement of the intervention indicated that the energy bar, sorghum bytes, multigrain sweet meal, and nutri-cookies were at the top of the beneficiaries’ preference list, especially among children (Table 2). The reason for the lower preferences for the sorghum meal and multigrain meal was mainly attributed to the spice levels; hence, the spice levels were adjusted accordingly, as per the beneficiaries’ feedback.

### 3.2. Impact of Intervention on Growth Parameters

A paired *t*-test was performed to assess the differences in the mean of the anthropometric measures in the treatment and control groups before the intervention (baseline) and post-intervention (endline) (Table 3). The treatment and control groups were similar and comparable in terms of their anthropometric measures prior to the intervention. The data analysis shows significant improvements (*p* < 0.01) in the average anthropometric measures at endline as compared to baseline in both the control and treatment groups. Importantly, the improvements in the anthropometric measures among the intervention group (column (DT-DC) (Table 3)) were significantly higher and can be attributed to the nutritional intervention. The intervention had the highest impact on improving the height of the children in the treatment group (average height at baseline 91.3 ± 8.72, at endline 104.1 ± 7.51, 3.2 cm more compared to the control group), followed by higher average weight gain (average weight at baseline 11.9 ± 2.26, at endline 15.5 ± 2.34, 1.68 kg greater in the treatment group relative to the control group) and MUAC (average MUAC at baseline 14.57 ± 1.38, at endline 15.6 ± 1.84, 0.33 cm more in the treatment group relative to the control group). The results indicate the success of the intervention in terms of its positive impacts, particularly on the height and weight of the children in the treatment group.

#### 3.2.1. Impact on Wasting (Weight-for-Height)

In the treatment group (n = 306), 7.2% of the children were severely wasted (WHZ < −3), and 20.8% were wasted (WHZ > −3 to <−2) at baseline. At endline, 72.7% of the severely wasted children had moved to the wasted category. In the control group (n = 81), 4.9% of the children were severely wasted, and 14.8% were wasted at baseline. At endline, there was a 25% increase in severely wasted children. A considerable reduction was seen in the treatment group in severe wasting and wasting, and there was a consequent increase in the normal category in terms of linear growth. The percentage changes (reduction or increase) in severe wasting, wasting, and normal were −72.7%, −51.6%, and +22.2% in the intervention group, respectively, while in the control group, there were 25% and 50% increases in severe wasting and wasting, respectively, and a consequent reduction in the normal category (−10.8%) (Table 4).

**Figure 4:**
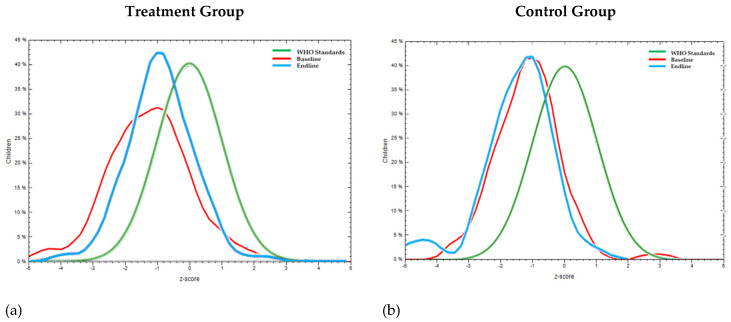
Comparison of Z-scores for wasting or weight-for-height (WHZ) curve with WHO standard curve at baseline and at endline (post-intervention using millet–pulse–groundnut-based formulations) of pre-school children in (**a**) treatment group (mean WHZ moved from −1.2 ± 1.3 to −0.9 ± 1) and (**b**) control group (mean WHZ from 1.2 ± 1 to −1.4 ± 1.1).

Overall, the impact of the intervention on the treatment population moved [(mean WHZ moved from −1.2 ± 1.3 to −0.9 ± 1 (Figure 4a)]. For the same period of the intervention, the control group (Figure 4b) showed a negative shift in mean WHZ from −1.2 ± 1 to −1.4 ± 1.1.

#### 3.2.2. Impact on Stunting (Height-for-Age)

In the treatment group, 20.8% of the children were severely stunted (HAZ < −3), and 25.7% were stunted (HAZ > −3 to <−2) at baseline. At endline, 87.5% of the severely stunted children had moved to the stunted category. In the control group, 17.3% of the children were severely stunted, and 24.7% were stunted at baseline. At endline, 64.3% of the severely stunted children in the control group had moved to the stunted category. There was a considerable reduction in severe stunting and stunting and a consequent increase in the normal category in terms of linear growth in both the treatment and control groups. However, the positive rate of change observed in the control group was less than that in the intervention group. There was a 73.8% increase in the number of children having normal growth in the intervention group compared to a 29.8% increase in children having normal growth in the control group at endline as compared to baseline (Table 4).

**Figure 5:**
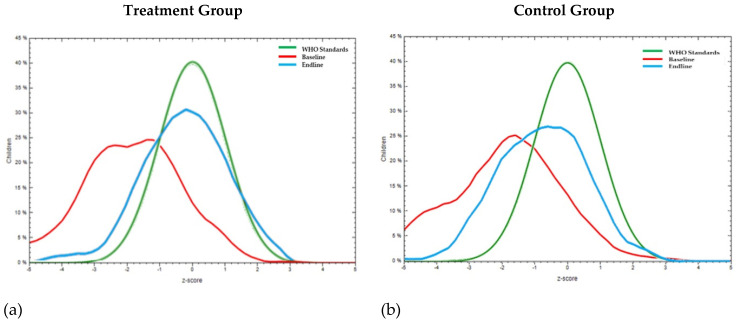
Comparison of Z-scores for stunting or height-for-age (HAZ) curve with WHO standard curve at baseline and at endline (post-intervention using millet–pulse–groundnut-based formulations) of pre-school children in (**a**) treatment group (mean HAZ moved from −1.8 ± 1.6 to −0.3 ± 1.3) and (**b**) control group (−2.0 ± 1.7 to −0.8 ± 1.3).

Overall, the impact of the intervention on the treatment population moved [(mean HAZ −1.8 ± 1.6 to −0.3 ± 1.3 for treatment (Figure 5a)]. For the same period, the control group (Figure 5b) showed a shift in HAZ from −2.0 ± 1.7 to −0.8 ± 1.3.

#### 3.2.3. Impact on Underweight (Weight-for-Age)

**Figure 6:**
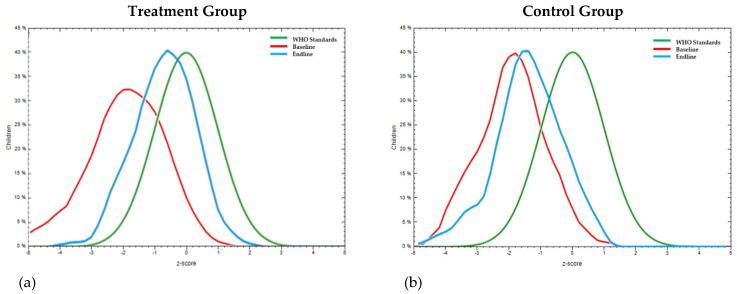
Comparison of Z-scores for underweight or weight-for-age (WAZ) curve with WHO standard curve at baseline and at endline (post-intervention using millet–pulse–groundnut-based formulations) of pre-school children in (**a**) treatment group (mean WAZ moved from −1.9 ± 1.2 to −0.7 ± 1) and (**b**) control group (mean WAZ from −1.9 ± 1 to −1.4 ± 1).

In the treatment group, 18.6% of the children were under the severe thinness category (WAZ < −3), and 27% were in the thinness category (WAZ > −3 to <−2) at baseline. At endline, 94.7% of the severe thinness category children had moved to the thinness category. In the control group, 8.6% of the children were in the severe thinness category, and 33.3% were in the thinness category at baseline. At endline, 14.3% of the severely thin children in the control group had moved to the thinness category. There was considerable reduction in the severe thinness and thinness categories, which consequently increased the number of children in the normal category in both of the groups. However, the positive rate of change observed in normal growth among children in the control group was less compared to children in the intervention group. There was a 66.5% increase in children having normal growth in the intervention group compared to a 25.5% increase in children having normal growth in the control group at endline compared to the baseline data (Table 4). Overall, the impact of the intervention on the treatment population moved [(mean WAZ −1.9 ± 1.2 to −0.7 ± 1 for treatment (Figure 6a)]. For the same period, the control group (Figure 6b) showed a shift in WAZ from −1.9 ± 1 to −1.4 ± 1.

### 3.3. Impact of Intervention on Haemoglobin (Hb) Levels

The pre-intervention and post-intervention measures were positively associated. Thus, post-intervention measurements can be predicted based on pre-intervention values; for instance, if the treatment (intervention) group is a better performer prior to intervention (pre), it tends to be the same after intervention (post), as seen in the following means Table 5.

The RM-ANOVA indicated a significant treatment × time interaction (*p* < 0.0001). A significant (Treat × Time) interaction effect implies that the Hb levels in the groups are changing over time and are changing in different ways. The main effect of the treatment was also significant, which implies that the control and the treatment groups differ significantly across the time points.

The change in Hb levels within the intervention group shifted significantly from 9.70 ± 0.14 g/dL to 11.08 ± 0.13 g/dL. This shift indicates that the previously moderately anaemic (Hb 7 to 9.9 g/dL) individuals in the treatment population transitioned to the normal Hb level category (Hb ≥ 11 g/dL) post-intervention. In comparison, for the control population, the post-intervention moved from moderately anaemic Hb levels from 8.36 ± 0.26 g/dL to 10.35 ± 0.24 g/dL to mildly anaemic levels (10 to 10.9 g/dL) [19]. This clearly shows the impact of consuming millet–pulse–groundnut-based food products on addressing the prevalent problem of anaemia among tribal children.

### 3.4. FGD Preference Rating of the Food Products

A total of 124 mothers and 26 AWC teachers were actively engaged in the FGDs and individual interviews, respectively. Mothers who participated in the outcome assessment were able to recollect all of the six types of nutritious millet–pulse–groundnut-based food products provided at the AWCs during the intervention. Based on the responses from the mothers, on a six-point Acceptance Rating (AR) scale (six indicating high acceptance and one indicating low acceptance) (Table 6), the energy bar was ranked as the most favoured product (AR = 4.7 ± 1.2), followed closely by nutri-cookies as the second (AR = 3.7 ± 1.3), sorghum bytes third (AR = 3.6 ± 1.2), and the multigrain sweet meal as the fourth choice (AR = 3.5 ± 1.8). The sorghum meal and multigrain meal had AR ratings of 2.0 ± 1.1 and 1.4 ± 1.3, respectively.

**Figure 7:**
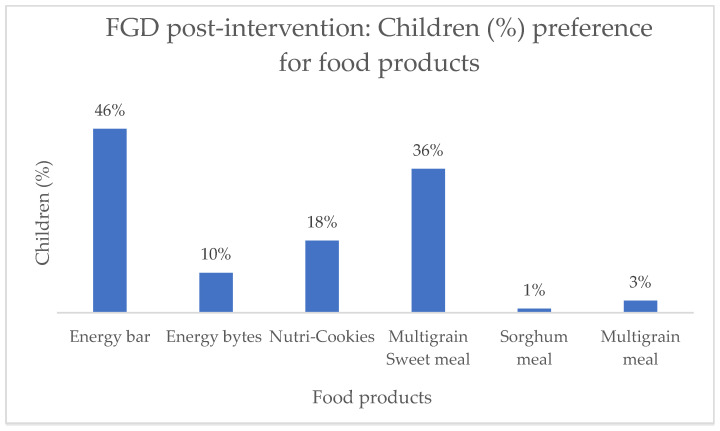
Preferences of food products by children from Bhadrachalam and Eturunagaram ITDAs.

Mothers from both ITDAs mentioned that the energy bar received the highest preference rate, with 46% of them stating that their children favoured it (Figure 7). Following closely, 36% of mothers rated the multigrain sweet meal as the next preferred option for their children. Only 3% and 1% of the mothers indicated that their children showed a preference for the multigrain savoury meal and the sorghum meal, respectively.

## 4. Discussion

The results of the current study demonstrate the beneficial effects of dietary interventions based on millet–pulse–groundnut products. Thus, this study was able to identify that the millet–pulse–groundnut-based food items provided during the intervention resulted in improvements in the health parameters under consideration of the treatment population.

This study verified that consuming a diet rich in millet and pulses helps combat undernutrition. This result aligns with past research conducted on millet consumption among adolescents in Karnataka State, India, which reported that the groups who received millet–pulse food showed significantly greater anthropometric measurement readings than the adolescents who were not part of the nutritional intervention [20]. A systematic review and meta-analysis study involving publications on health impacts of millet-based diets in comparison to consumption of only rice-based diets showed evidence of the health benefits of consuming millets. Significant effects (*p* < 0.05) were indicated on mean height (eight publications) (+28.2%), weight (nine publications) (+26%), mid-upper-arm circumference (five publications) (+39%), and chest circumference (five publications) (+37%) in comparison to regular rice-based diets.

In another study conducted among adolescent girls, millet-based foods had a significant impact on weight and BMI, and they also contributed to improvements in Hb levels [21]. Post-intervention data provided evidence that the mean weight improved from 26.77 ± 1.4 kg to 26.92 ± 2.1 kg, the mean BMI increased from 16.4 ± 2.5 kg/m^2^ to 17.3 ± 2.2 kg/m^2^, and haemoglobin levels increased from 8.40 ± 0.9 g/dL to 8.45 ± 0.6 g/dL.

The positive effect on Hb levels in the current study findings are in line with other studies [21,22] showing that supplementation with iron-rich millet-based foods has a positive impact on haemoglobin levels and reduces the prevalence of anaemia.

Consumer acceptability has also been garnered for iron-rich snacks formulated using under-utilised millet grains [23]. A “Multi-millet health mix” has been proven to have a positive effect on increasing the anthropometric indices of children [24]. Our study further corroborates the acceptance of millet–pulse–groundnut-based RTE and RTC products among the target beneficiaries, leading to similar positive impacts on anthropometric indices as well as haemoglobin levels.

Among the six recipes used for the intervention, four were generally liked by all of the children (Table 2). However, the multigrain savoury meal and sorghum meal were identified to have mixed responses among children, which was attributed mainly to the individual’s preference for salt, spices, etc. Despite an initial sensory study to fine-tune the formulations, the said products elicited mixed responses, especially among children. This could be attributed to the changes in taste preferences in children of growing age. Further, mothers also informed that the meals were sometimes saltier. The possible reasons may have involved not utilising the pack containing the composite meal for a single serving or not shaking the packet well before using, resulting in salt deposits at the bottom of the packets. The AWC teachers also mentioned that the sorghum meal and the multigrain meal were the least popular products among the cooks because these two products took comparatively more time to cook. The most liked recipes were the sweet items, especially the energy bar and multigrain sweet meal, because of the taste preference. Repeated demands for the products provide testimony to the fact that the sweet products were most preferred by the children. This highlights the necessity for ongoing product evaluation and modification in the formulations when it comes to school meals, because acceptance among children may change for various reasons, such as changes in children’s tastes or time taken for meal preparation by the community workers.

Mothers exhibited an improved understanding of the benefits that their children accrue from consumption of millets and pulses, which was clear from the FGDs (Table S1). The mothers stated that they had attempted to replicate some of the products provided at the AWCs in their homes. Despite comprehending these incremental benefits of millets, mothers outlined and expressed concerns regarding the longer cooking time of millet meals compared to rice. This implies that there is scope for behavioural change communication interventions, complemented with the sharing of nutritious recipes and their preparation protocols with the mothers in their local languages. The government could allocate further funds towards research and development and pilot testing of easy cooking and inclusive millet-based recipes. Further, to facilitate behavioural change towards dietary diversity in rice-based meal consumption patterns and to provide ease of work for the AWC staff workers in terms of the number of preparations, interventions must be integrated to provide millets in the mid-day meal (MDM) rather than as an additional meal with a rice-based mid-day meal. The Uttar Pradesh Government has taken steps in this direction by deciding to incorporate millets in the form of ‘Bajra khichdi’ in the MDM provided to students [25].

A similar discussion with AWC teachers revealed that the prevalent practices and preference for rice production were catalysed by water availability. Further, they also highlighted that millet production involved high risks and high production costs (labour costs for bird scaring, anti-bird nets to prevent losses by birds and animals, which are comparatively greater in tribal-dense forest areas). The above statements indicate the need to further intensify efforts by the government to encourage diversified farming systems in the country through appropriate incentives and better market linkages, along with promoting current policies supporting millet production. The PDS could purchase more millets through the Food Corporation of India (FCI) across the country and distribute them for consumption through the Fair Price Shops (FPSs) ration and school feeding programmes, creating enhanced demand and markets for millets. The inclusion of millets in the PDS policy at an affordable price, along with sensitisation about the health benefits of millet consumption, will result in sustained “demand pull” for millets.

Thus, apart from the nutritional benefits that are shown to be positively correlated with the intervention, this study also upholds significant implications for formulating appropriate polices to promote “demand driven” production of millets and related value-added products. Thus, the results from the current millet–pulse–groundnut-based nutritional intervention study confirm that improved growth and nourishment among the tribal children can be brought about by diversifying the diets to include millets, pulses, and oilseeds. Apart from the statistical results, the conclusion is also empirically corroborated by the fact that AWC teachers reported a noticeable change in health and cognitive status among the children during the intervention period (Table S2). 

## 5. Conclusions

It is noteworthy that the micronutrient demand is still unmet through natural diets, even in developed countries. Thus, concerted efforts to strengthen existing food systems in response to climate crises and malnutrition are the need of the hour. In this regard, incorporating climate-resilient nutritious crops such as millets into diet systems to create resilient and sustainable agri-food systems is one solution. Interventions such as the one described in this study are one of the solutions by which millets can gain importance in daily diets. The intervention was successful in improving the nutritional status of the children monitored, from being categorised as moderately acutely malnourished to normal. Thus, this study provides evidence to suggest that interventions that are co-developed and co-designed with the relevant target population and stakeholders/community participation, and which undergo timely administration, have high potential for achieving the desired impacts. Such interventions are likely to drive behavioural changes in consumption patterns. The International Year of Millets 2023 (IYM23) has provided us with the right opportunity to further scale up such initiatives for the future. IYM23 has attracted the global community by showcasing millets as a potential solution for achieving climate-resilient and sustainable food systems and thus achieving nutritional security. The mission has paved the way for global collaborative R&D support for the generation of a strong evidence base towards further understanding and promoting awareness on the health benefits of millets. Initiatives such as these are suitable and efficient responses to combat the triple burden of malnutrition and also contribute to the growing evidence base of the nutritional benefits of millets.

The authors recognise the limitations of this study in the following areas:

1. Operationalisation of the research study: With the onset of the COVID-19 pandemic, the restrictions on movements and closure of the AWCs necessitated change in the product formats (from RTC to RTE), as the food products needed to be distributed as take-home rations (THRs) directly to the beneficiary households. The challenges posed by the pandemic and other natural disasters, such as floods in the intervention areas, caused intermittent breakdowns in the distribution of the food products, resulting in an extension of the project period from 12 to 18 months.
2. Reduction in sample size: The tribal population are migrants by their cultural profile, and some of the participants migrated away from the project location within the course of the project duration. Some of the children moved from Anganwadis (daycare feeding centres) to primary schools, so they were unavailable during the endline analysis. This reduced our sample size.
3. Lower number of individuals in the control group: The issues with our sample size also spilled over into the number in the control group, which was less than one-third of the treatment group. Thus, the accuracy of the results may be doubted.
4. No gender-wise disaggregated results: Disaggregating the small sample further by gender would have made the sample too small to arrive at precise and meaningful results. Thus, we could not conduct any gender-disaggregated analyses across the treatment and control groups.
5. Localised results: This study was conducted only in two regions of a province in India; thus, the results cannot be generalised.

Despite these limitations, this study makes an interesting contribution to the literature by providing empirical evidence on the effectiveness of millet–pulse–groundnut-based food products in improving the diets of tribal children. This study is unique among existing research in the way it targets improving the nutritional levels of tribal populations by leveraging climate-resilient, nutritious millets.

Though this study was localised in its effects and was conducted in some of the tribal regions of Telangana, the scaling up and adaptation of such initiatives in India and other developing countries, where millets and pulses are common and where malnutrition is widespread, would ensure significant progress towards building a food-secure society.

## Figures and Tables

**Table 1:** Nutrient profiles of the products provided during the intervention.

Nutrition Parameters Tested (per 100 g of the Product)	Ready-to-Cook			Ready-to-Eat		
	Sorghum Meal	Multigrain Meal	Multigrain Sweet Meal	Nutri-Cookies	Energy Bar	Sorghum Bytes
Energy (kcal)	362.1	350.4	446.1	472.4	509.1	462.9
Protein (g)	12.3	14.6	4.8	5.8	16.0	9.0
Fat (g)	4.8	2.2	15.1	25.2	24.9	24.5
Carbohydrate (g)	65.4	66.0	75.0	55.7	54.5	51.5
Calcium (mg)	47.1	70.0	47.0	71.2	188.5	31.9
Iron (mg)	4.5	4.3	2.9	1.9	5.6	2.3

**Table 2:** Results of acceptability study conducted using millet–pulse–groundnut-based products (pre-intervention) among the pre-school children.

Product Name	Children (%) Accepting a Product (Eturunagaram)	Children (%) Accepting a Product (Bhadrachalam)
Sorghum meal	95	80
Multigrain meal	80.7	93
Multigrain sweet meal	85.5	100
Energy bar	100	100
Sorghum bytes	96.8	100
Nutri-cookies	85.5	100

**Table 3:** Differences in mean anthropometric measures.

Anthropometry	Treatment						Control						
	Baseline	Std. Dev	Endline	Std. Dev	*p*-Value	Difference (DT) ^#^	Baseline	Std. Dev	Endline	Std. Dev	*p*-Value	Difference (DC) ^#^	DT-DC
Height (in cm)	91.3	8.72	104.1	7.51	0.000 ***	12.8	90.1	10.36	99.7	9.13	0.000 ***	9.6	3.2
Weight (in kg)	11.9	2.26	15.5	2.34	0.000 ***	3.58	11.7	2.27	13.6	2.53	0.000 ***	1.9	1.68
MUAC (in cm)	14.57	1.38	15.6	1.84	0.000 ***	0.99	15.1	1.02	15.7	2.08	0.007 ***	0.66	0.33

*** *p* < 0.01, Note that all differences are significant at 1% for paired *t*-test and were determined in Stata. **^#^** Column “Difference” indicates the differences in baseline and endline measurements. Column “DT-DC” is the difference between difference (T) and (C).

**Table 4:** The change (% change and % difference at endline, in relation to baseline) of children in the categories of wasting, stunting, and underweight between intervention and control groups of pre-school children (3 to 6 years).

Status of Treatment Group vs. Control Group	Wasting (%)			Stunting (%)			Underweight (%)		
	Severe Wasted (WHZ < −3)	Wasted (WHZ > −3 to <−2)	Normal	Severe Stunted (HAZ < −3)	Stunted (HAZ > −3 to <−2)	Normal	Severe Thinness (WAZ < −3)	Thinness (WAZ > −3 to <−2)	Normal
Treatment Group									
Baseline	7.2	20.8	72.0	20.8	25.7	53.4	18.6	27.0	54.4
Endline	2.0	10.1	87.9	2.6	4.6	92.8	1.0	8.5	90.6
B. Control Group									
Baseline	4.9	14.8	80.2	17.3	24.7	58.0	8.6	33.3	58.0
Endline	6.2	22.2	71.6	6.2	18.5	75.3	7.4	19.8	72.8
Difference (%) Treatment vs. Control	% Difference in wasting			% Difference in stunting			% Difference in underweight		
Treatment	−72.7	−51.6	22.2	−87.5	−82.3	+73.8	−94.7	−68.7	+66.5
Control	+25.0	+50.0	−10.8	−64.3	−25.0	+29.8	−14.3	−40.7	+25.5

**Table 5:** Group × time means.

Study Groups	Time	Estimate (g/dL)	Standard	Mean Differences (Pre and Post)	
			Error	*t*-Value	*p*-Value
Control	Pre	8.3696	0.2607	6.33	<0.0001
Control	Post	10.3506	0.2401		
Treatment	Pre	9.7030	0.1379	8.36	<0.0001
Treatment	Post	11.0794	0.1238		

**Table 6:** Acceptance score of the products provided in both ITDAs.

Food Products	Mean of Acceptance Rating for the Products *	Ranking
Energy Bar	4.7 ± 1.2	1
Nutri-Cookies	3.7 ± 1.3	2
Sorghum Bytes	3.6 ± 1.2	3
Multigrain Sweet meal	3.5 ± 1.8	4
Sorghum meal	2.0 ± 1.1	5
Multigrain meal	1.4 ± 1.3	6

* Represents mean of acceptance rating from both Bhadrachalam and Eturunagaram ITDAs.

## Data Availability

Data are contained within the article and Supplementary Materials.

## Supplementary Materials

The following supporting information can be downloaded at: https://www.mdpi.com/article/10.3390/nu16060819/s1, Table S1: Feedback form for product acceptability study; Table S2: NFHS-4 data of Khammam district (ITDA Bhadrachalam) and Warangal district (now Jayashankar Bhupalpally district) (ITDA Eturunagaram) of Telangana; Figure S1: percentage of Recommended dietary allowance (RDA) achieved through different food products (ICDS and giri poshana); Questionnaire S1: Questionnaire for Focussed Group Discussions-Giri Poshana post intervention.
