# Supplementary material for: Effectiveness of Millet–Pulse–Groundnut Based Formulations in Improving the Growth of Pre-School Tribal Children in Telangana State, India
Source: Nutrients. 2024 Mar 13;16(6):819. doi: 10.3390/nu16060819 (PMC10974459; doi:10.3390/nu16060819)
Supplement: Supplementary file 1 [file nutrients-16-00819-s001.zip › nutrients-2839355-supplementary.pdf]

Supplementary materials for the manuscript "Effectiveness of Millet–Pulse–Groundnut Based Formulations in Improving the Growth of Pre-School Tribal Children in Telangana State, India"

Table S1: Feedback form for product acceptability study

(pre-intervention study to understand the acceptability towards millet-pulse-groundnut based products)

**Name :**					**Age:**		**Place:**
**S. No.**	**Questions**	**Food products**					
		**Sorghum Meal**	**Multigrain meal**	**Multigrain sweet meal**	**Nutri-cookies**	**Energy bar**	**Sorghum bytes**
1	How do you rate the product (rating)						
2	When do you like to eat this product Morning (M)/Afternoon (A)/Evening (E)/Night (N)						
3	Do you think it is healthy? Yes (Y) / No (N)						
4	Do you recommend this product to your children and other family members Yes (Y) / No (N)						
5	How do you rate the product in terms of following:						
	1. Saltiness						
	2. Spiciness						
	3. Sweetness						
6	Do you have any other suggestions?						

Rating: 1 = dislike very much, 2 = dislike slightly, 3 = neither like nor dislike, 4 = like slightly, 5 = like very much

Table S2 : NFHS-4 data of Khammam district (ITDA Bhadrachalam) and Warangal district (now Jayashankar Bhupalpally district) (ITDA Eturunagaram) of Telangana

Indicators	NFHS-4 (erstwhile Khammam District)	NFHS-4 (erstwhile Warangal district)
Children under 5 years who are underweight (weight-for-age) (%)	22.2	29.1
Children under 5 years who are stunted (height-for-age) (%)	26.5	26.6
Children under 5 years who are wasted (weight-for-height) (%)	13.7	16.6
Children aged 6-59 months who are anemic (<11.0 g/dl) (%)	72.2	63

Source: **https://rchiips.org/nfhs/**

The graph below shows %RDA achieved through Integrated Child Development Services (ICDS) mid-day meal (rice+dal+oil+veg), egg, nutri-snacks along with Giri Poshana food (*three Giri Poshana combinations). The %RDA of energy achieved through Giri Poshana food is 26.6% (average of three food combinations) makes supplementary nutrition for the children with the regular %RDA through existing ICDS meal (27%).

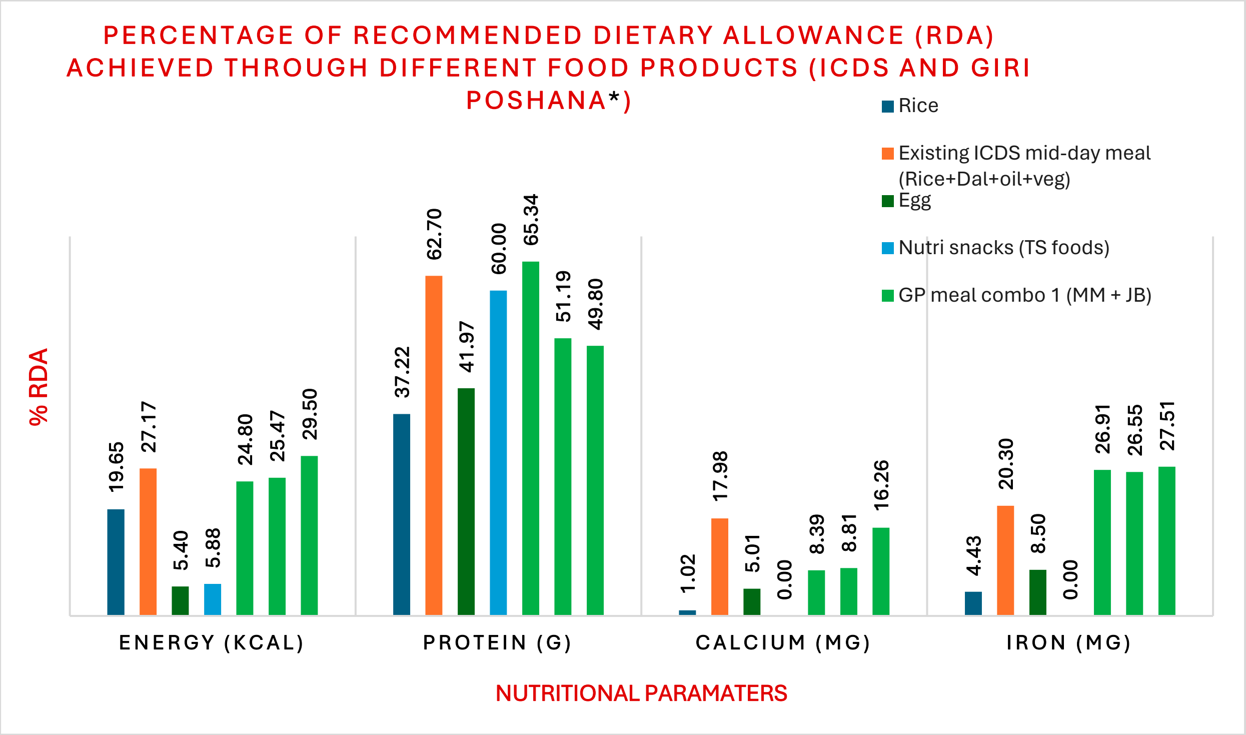

Figure S1: percentage of Recommended dietary allowance (RDA) achieved through different food products (ICDS and giri poshana)

**Giri Poshana meal combination 1: Multigrain meal + Jowar bytes (provided on Monday & Thursday)*
Giri Poshana meal combination 2: Jowar meal + Nutri -cookies (provided on Tuesday & Friday)
Giri Poshana meal combination 3: Multigrain sweet meal + Energy bar (provided on Wednesday & Saturday)

Giri Poshana meals (millet-pulse-groundnut based food products) are designed in ready to cook formats, which are very convenient to cook and reduces drudgery of anganwadi workers in cooking and saves lot of time. The millets used in the food product formulations are 'malted' to enhance digestibility of carbohydrate/proteins and ensure reduction in anti-nutrients, leading to enhanced bioavailability of micronutrients. Malting Simple processing technique which involves soaking, germination and drying and can help tackling malnutrition by improving protein digestibility and the bioavailability of the minerals. Most importantly, the products were designed only to diversify and supplement the existing diets of the target population and were not fortified with additional micronutrients to immediately address nutritional deficiencies.

The Anganwadi teachers were sensitized on, cooking methods for the RTC products, serving portions for each, the nutritional benefits along with awareness on basic hygiene practices to be followed during food preparation and handling.

S1. Questionnaire for Focussed Group Discussions- Giri Poshana post intervention

I. Category: Beneficiary - children (3-6yrs)

1. **Giri Poshana Intervention structure**

   a. **Breakfast**

- What are the food products consumed for breakfast as part of the GP project?
- Were you satisfied with the GP food given as breakfast?
- Do you think that the GP breakfast should have been given during some other time? Y/N; if yes, suggest timings
- Was it convenient for you to go the Anganwadi center and have breakfast? Y/N If N why?
- What breakfast were you consuming before the intervention?
- What breakfast are you consuming now after the intervention is over?

   b. **Afternoon snacks (3-4pm)**

- What are the food products consumed for snacks as part of the GP project?
- Were you satisfied with the GP food given as snacks?
- Was it convenient for you to go the Anganwadi center and have snacks? Y/N If N why?
- What snacks were you consuming before the intervention?

- What snacks are you consuming now after the intervention is over?

c. **Portion sufficiency**

- Do you eat anything additionally for breakfast at home?

- Is the quantity given for breakfast filling? Y/N; If No, suggest quantity (less/more)

- Is the quantity given for snacks enough? Y/N; If No, suggest quantity (less/more)

d. **Frequency of feeding**

- Was there continuity in GP food consumption? Y/N; If No, what is the reason?

e. **Taste and flavor**

- Did you like the taste/flavor of the GP food products

f. **Behavioral change**

- Did you find any difference on Sundays when you don’t eat the GP food? Y/N; If Y, reason?

2. **Attendance**

- Did GP food encouraged you to go to anganwadi center every day?

3. **Consumer preference:**

   Rank the food products according to the preference (1 – most preferred to 5 – least preferred)

   1. Jowar meal ____________________
   2. Multigrain meal ____________________
   3. Multigrain sweet meal ____________________
   4. Nutri-cookies ____________________
   5. Energy bar (chikki) ____________________
   6. Jowar bytes ____________________

   Any other comments:

II. Category: Parents of beneficiaries (children 3-6yrs)

1. **Giri Poshana Intervention structure**
   - What are the GP food products consumed for breakfast and snacks by your children as part of the GP project?
   - Was it convenient to send your children to the Anganwadi center to have GP food? Y/N If N why?
   - Do children eat anything additional for breakfast at home?
   - Are children hungry after coming home from Anganwadi center?
   - What food (breakfast and snacks) were children consuming before the intervention?
   - What food (breakfast and snacks) are children consuming now after the intervention is over?

- Were your children happy/losing interest to consume GP food?
  If happy, which GP food product they liked most?
  If losing interest, Reason?
- Have children ever mentioned the GP food products consumed at Anganwadi?
- If yes, what are the products they have mentioned.
- Did children like the taste/flavor of the GP food products
- Do you think GP food is nutritious and improves kids' health?
- Are you willing to pay and purchase from the market, if its available?

2. Behavioral change

a. **Keenness to eat** -

- How interested the children were towards eating the GP food
- Do the children ask for GP food during Sundays or after the intervention?

b. Hygiene

- Does your child practice hand wash at home?

c. Improvement in their attention

- Have you noticed any change in childs' attentiveness at home?

- Were they dull before? Any change now?

3. **Physical change**
   - Any visible change in height/weight of children?

   - Improvement in physical activity

   - Frequency of sickness? Any change after GP food

   - Any change in time taken for recovery from illness?

4. **Attendance**
   - Does GP food play any role in encouraging the children to attend AWC regularly?

5. **Awareness on millets**

   a. **Do you know millets is considered highly nutritious and good for health? Yes / No**

   b. **What are the qualities you know about millets and millet based products?**
      - Provides higher energy
      - High in nutrition
      - Increases appetite
      - Has higher proteins
      - Give more strength
      - Good for weight loss
      - Good for diabetics
      - Good for blood pressure management
      - Good for immunity
      - I don't know

c. **Presently are you consuming any millet products in your family?** Yes/ No

____

____

d. **What kind of millet products do you consume in your family?**
   - Traditional products
   - Value added product like Biscuits/ Flakes/ Noodles/ Dalia/ laddu/ Pasta/ Vermicelli

____

____

e. **Do you think there is an increase or decrease in the consumption of traditional millet products?**
   - Increase (If yes please provide response to Q f)
   - Decrease (If yes please provide response to Q g)

f. **What are the main reasons for increasing consumption of traditional millet products? Rank them (**1 – most preferred to 5 – least preferred)
   - Better taste
   - Easy availability
   - Easy to prepare
   - Healthier/ nutritious food and provide more immunity than other Value-added products
   - Value for money
   - Community influence
   - Good for children and old age people
   - Increased awareness on their nutritional quality
   - Recommended by doctor/ nutritionist
   - Ready to cook product
   - Ready to eat product
   - Good packaging

g. **What are the main reasons for decreasing consumption of traditional millet products? Rank them** (1 – most preferred to 5 – least preferred)
   - Better taste
   - Easy availability
   - Easy to prepare
   - Healthier/ nutritious food and provide more immunity than other Value-added products
   - Value for money
   - Community influence

- Good for children and old age people
- Increased awareness on their nutritional quality
- Recommended by doctor/ nutritionist
- Ready to cook product
- Ready to eat product
- Good packaging

6. **Regular dietary pattern of the beneficiary**

____

____

III.Category: Key informant interview with the Anganwadi Teacher from the intervention Anganwadi schools

1. **Giri Poshana Intervention structure**

- Were beneficiaries satisfied with the GP food given as breakfast and snacks

  ____

  ____

- Do you think that the GP breakfast should have been given during some other time? Y/N; if Yes, suggest timings

  ____

  ____

- Is the quantity given for breakfast and snacks, filling? Y/N; If No, suggest quantity (less/more)

  ____

  ____

- How often was the GP food distributed to the anganwadi centre?

  ____

  ____

- Was the GP food received on time? (Regular/Irregular) ? If irregular, why?

  ____

  ____

- Did beneficiaries like the taste/flavor of the food products?

  ____

  ____

- What is the meal plan that is being followed in Anganwadi centre? Details of other (Non- GP) foods?

  ____

  ____

2. **Consumer preference:**
   - Rank the food products according to the preference (1 – most preferred to 5 – least preferred)

     Jowar meal ____________________
     Multigrain meal ____________________
     Multigrain sweet meal ____________________
     Nutri-cookies ____________________
     Energy bar (chikki) ____________________
     Jowar bytes ____________________

   Any other comments:

   ____

   ____

3. **Behavioral change**

   a. Keenness to eat

   - How interested the beneficiaries were towards eating the food Do the children ask for GP food after the intervention?

     ____

     ____

   b. Hygiene

   - Do they practice hand wash at anganwadi center?

     ____

     ____

   c. Improvement in their attention

   - Have you noticed any change in their attentiveness in the class/home?

     ____

     ____

   - Were they dull before? Any change now?

d. **Performance in school/AWC**

- Are they punctual to AWC?
- Do children follow timings?

4. **Physical change**

- Any visible change in height and weight of children?
- Improvement in physical activity
- Frequency of sickness? Any change after GP food
- Any change in time taken for recovery from illness?

5. **Attendance**

- Does GP food play any role in encouraging the children/women to attend AWC regularly?
- Did all beneficiaries show willingness to consume GP food? did they consume it regularly?
- Did attendance of the beneficiaries, especially children improve during the GP project? Y/N. If Y what was the reason?

- Have all beneficiaries consumed regular AWC food (other than GP)?

- What do you think the reason for unavailability of beneficiaries during endline survey?

6. **Did you face any other challenges in implementation of GP project? Please specify**
